# Three-Year Results of the GORE VIABAHN Endoprosthesis in the Superficial Femoral Artery for In-Stent Restenosis

**DOI:** 10.1016/j.jscai.2023.100598

**Published:** 2023-02-20

**Authors:** Peter Soukas, Matthew Becker, Karl Stark, Gunnar Tepe, RELINE MAX Investigators

**Affiliations:** a Lifespan Cardiovascular Institute, the Miriam Hospital, Alpert School of Medicine of Brown University, Providence, Rhode Island; b Lake Erie College of Medicine Heart and Vascular Institute, Cardiovascular Medicine, Interventional Cardiology and Cardiac Catheterization Laboratory, Ambulatory Surgical Vascular Institute, Erie, Pennsylvania; c Midwest Aortic and Vascular Institute, University of Missouri at Kansas City School of Medicine, University of Health Sciences, Kansas City, Missouri; d Department of Diagnostic and Interventional Radiology, RoMed Clinic Rosenheim, Germany

**Keywords:** Endovascular therapy, in-stent restenosis, peripheral arterial disease, stent-graft, superficial femoral artery

## Abstract

**Background:**

The study objective was to assess the postmarket safety and effectiveness of the GORE VIABHAN endoprosthesis with heparin bioactive surface for the treatment of in-stent restenosis (ISR) of the superficial femoral artery (SFA).

**Methods:**

A prospective, single-arm, international study enrolled patients at 23 sites from October 2015 to April 2018. Patients with ≥50% ISR or occlusions in the SFA, Rutherford categories 2-5, and at least 1 patent runoff vessel were eligible. The primary effectiveness endpoint was primary patency at 12 months. The primary safety endpoint was the rate of device- or procedure related serious adverse events at 30 days.

**Results:**

One hundred and eight patients were enrolled, and 86 were included for analysis through 3 years (mean age, 70.0 ± 10.4 years; 48.8% female). The mean core lab reported lesion length was 12.4 ± 6.92 cm (29.1% occlusions); 10.5% presented with chronic limb-threatening ischemia, and 81.9% of lesions were Tosaka II and II. Acute procedural success was 98.8%. Freedom from device- or procedure-related SAE was 96.5% through 30 days. At 1-year, primary, primary-assisted, and secondary patency rates were 74.7%, 80.4%, and 98.4%, respectively. Freedom from target lesion revascularization was 84.8%, 74.6%, and 65.0% at 1, 2, and 3 years, respectively. Per core laboratory assessment, no major amputations or device failures occurred through 3 years. At 3 years, 80.4% of patients had ≥ 1 Rutherford category improvement.

**Conclusions:**

The VIABAHN endoprosthesis is a safe and effective treatment for long and complex lesions in the SFA through 3 years.

## Highlights

• Long and complex femoropopliteal in-stent restenosis is challenging to treat
• To our knowledge, first report of long-term 36-month outcomes in the postapproval study
• Food and Drug Administration-approved stenting option for femoropopliteal in-stent restenosis in the United States
• Safe and effective as a treatment for in-stent restenosis lesions

## Central Illustration

**Figure:**
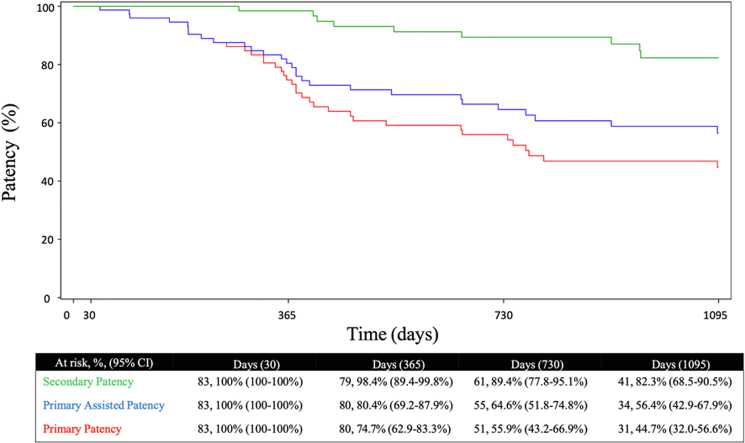

## Introduction

For complex in-stent restenosis (ISR) femoropopliteal (FP) lesions, especially in long or otherwise complex Tosaka II/III lesions,^1^ stents may be used as either primary therapy or as “bail out” after significant flow-limiting dissection or recoil. Unfortunately, FP-ISR remains common following stenting of the FP segment with bare metal stents.^2^ Given the long-standing correlation between long lesions and restenosis,^3^ ISR is arguably one of the most challenging patient populations to treat.^4^, ^5^, ^6^

Because of the challenges associated with ISR, few studies have looked at long lesions and >1-year time points. The Drug-Eluting Balloon in Peripheral Intervention for In-Stent Restenosis (DEBATE-ISR) study observed no differences in target lesion revascularization (TLR) between drug-eluting balloons (DEBs) and percutaneous transluminal angioplasty (PTA) at 3 years. However, Tosaka class III lesions were associated with increased TLR, regardless of DEBs or PTA use.^7^ The Zilver PTX Drug-Eluting Peripheral Stent System (Cook Medical) had a 12-month patency outcome of 78.8%^8^; however, it is not approved for lesion lengths >14 cm. The EXCImer laser is the only other device that is approved for the treatment of ISR. The EXCImer Laser Randomized Controlled Study for Treatment of FemoropopliTEal In-Stent Restenosis (EXCITE ISR) trial results established that this laser atherectomy catheter can be used as an adjunct therapy for PTA in ISR.^9^

The GORE VIABAHN endoprosthesis with heparin bioactive surface (VIABAHN endoprosthesis; W.L. Gore & Associates) is currently the only Food and Drug Administration-approved stenting option for FP-ISR in the United States. The VIABAHN endoprosthesis mechanism for the prevention of restenosis relies on its design of a heparin-coated expanded polytetrafluoroethylene (ePTFE) graft and a nitinol stent frame. Although a covered stent helps exclude endothelial cell growth into the device lumen, the CARMEDA BioActive Surface heparin surface on the VIABAHN endoprosthesis has been shown to lower platelet activation, reduce thrombus formation, and reduce neointimal hyperplasia. In addition, a prospective, randomized controlled trial (RELINE) assessed the safety and efficacy of the VIABAHN endoprosthesis as a treatment for ISR superficial femoral artery (SFA) lesions in a European patient population. It reported significantly higher primary patency (PP) compared with PTA at 12 months (74.8% vs 28.0%, *P* < .001), resulting in Food and Drug Administration approval for this indication in lesions up to 27 cm in 2014.^10^ The postmarket study data presented here were collected to confirm the safety and effectiveness of the VIABAHN endoprosthesis as a treatment for SFA-ISR in a largely US patient population. This is the first report of 36-month outcomes in the postapproval study of the GORE VIABAHN endoprosthesis for the treatment of ISR in the SFA (RELINE MAX).

## Materials and methods

### Study design

A prospective, single-arm, postmarket study was conducted at 23 sites (20 US, 3 outside the US, with 1 site each in Italy, Germany, and Sweden). The trial was conducted in accordance with the Declaration of Helsinki and Good Clinical Practice regulations (ClinicalTrials.gov identifier NCT02542267). All sites provided institutional review board or ethics committee approval of the study protocol, and all patients provided informed consent prior to study enrollment. Patients with symptomatic peripheral arterial disease (Rutherford categories 2-5) and ≥50% ISR and/or occlusion within or adjacent to a previously implanted (≥30 days) bare metal stent in the SFA (ending at least 1 cm above the intercondylar notch) were considered eligible for enrollment. A full list of study inclusion and exclusion criteria is available in Table 1.

**Table 1:** RELINE MAX inclusion and exclusion criteria.

**Inclusion criteria (Clinical)**
1. Patient is ≥18 years old at the time of informed consent signature
2. Patient is willing to give written informed consent
3. Patient has a previously implanted (>30 d) non-covered stent(s) located in the superficial femoral artery
4. Patient has lifestyle limiting claudication, resting leg pain, or minor tissue loss (Rutherford category 2-5)
5. Patient demonstrates an ABI ≤ 0.9. If ABI > 0.9 or not measurable, the patient is eligible for the study if the toe-brachial index is ≤0.5
6. Patient is male, infertile female, or female of childbearing potential with a negative β-hCG pregnancy test within 7 d of the index procedure
7. Patient can comply with protocol requirements, including follow-up visits
**Inclusion criteria (angiographic)**
1. Patient has ≥50% in-stent restenosis and/or occlusion in a previously implanted (>30 d) noncovered stent(s) located in the superficial femoral artery defined as beginning at least 1 cm below the origin of the profunda femoris artery and ending at least 1 cm above the intercondylar notch
2. Patient has a maximum total lesion length of 270 mm, consisting of in-stent and adjacent occlusive disease
3. Patient has a minimum of 1 cm of non-stenosed vessel both proximal and distal to the target lesion(s)
4. Patient has a reference vessel diameter between 4.0 and 6.5 mm
5. The guidewire and delivery system must cross the target lesion(s) intraluminally
6. Patient has a patent popliteal artery (<50% stenosis) distal to the target vessel
7. Patient has at least 1 patent infrapopliteal runoff vessel (<50% stenosis) not requiring intervention
8. Angioplasty balloon can be fully expanded in the target lesion during pretreatment step
**Exclusion criteria (clinical)**
1. Patient has a known allergy to stent-graft components (nickel-titanium or ePTFE)
2. Patient has a known allergy to contrast media that cannot be adequately premedicated prior to the study procedure
3. Patients with known hypersensitivity to heparin, including those patients who have had a previous incidence of heparin-induced thrombocytopenia type II
4. Patient has a known intolerance to anticoagulation or antiplatelet therapy
5. Patient has an uncorrected bleeding disorder (platelet count of <80,000/μL)
6. Patient has any known coagulation disorder, including hypercoagulability
7. Patient has severe chronic renal insufficiency (creatinine level of ≥2.5 mg/dL) within 30 d prior to study procedure unless currently on hemodialysis
8. Patient has septicemia or uncontrolled infection
9. The patient has any planned surgical intervention/procedure within 30 d of the study procedure
10. Patient has major distal amputation (above the transmetatarsal) in the study or non-study limb
11. Patient has prior ipsilateral femoral artery bypass
12. Patient has severe medical co-morbidities (untreated CAD/CHF, severe COPD, metastatic malignancy, dementia, etc.) or other medical conditions that would preclude compliance with the study protocol or 3-y life expectancy
13. Patient has severe ipsilateral common or deep femoral disease requiring intervention
14. Patient has any previous surgery in the target vessel
15. Patient has had vascular access via the lower extremities within 30 d of the index procedure
16. Patient has had previous target vessel in-stent restenosis treated by relining with another stent
17. Patient is currently participating in another clinical research trial unless approved by Sponsor
**Exclusion criteria (angiographic)**
1. The patient has untreated flow-limiting aortoiliac stenotic disease
2. Patient has an aneurysm adjacent to the target lesion(s)
3. Patient has a perioperative unsuccessful ipsilateral percutaneous vascular procedure to treat inflow prior to enrollment during the index study procedure
4. Patient has a femoral or popliteal aneurysm located in the target vessel
5. Patient has nonatherosclerotic disease resulting in occlusion (eg, embolism, Buerger disease, and vasculitis)
6. Patient has angiographic evidence of intraarterial thrombus or atheroembolism from inflow treatment
7. Patient has target lesion access not performed by transfemoral approach

ABI, ankle-brachial index; CAD, coronary artery disease; CHF, congestive heart failure; COPD, chronic obstructive pulmonary disease; ePTFE, expanded polyterafluoroethylene; hCG, humanchorionicgonadotropin.

### Study procedure

Ipsilateral antegrade or contralateral retrograde femoral artery access was performed. Intraluminal passage of the guidewire through the target lesion was required. Final patient eligibility was determined with a procedural angiographic assessment of the target lesion. Baseline images (angiogram or x-ray) of the previously implanted stent were captured and evaluated by an independent core laboratory (Yale Cardiovascular Research Group), along with procedural angiographic measurements and procedural and follow-up x-ray imaging. Target lesion predilation with full expansion of the balloon within the lesion was performed prior to stent implantation, with marking of the margins of predilation to ensure complete stent coverage of all predilated areas with the VIABAHN endoprosthesis. Per protocol, the endoprosthesis extended a minimum of 1 cm proximally and distally into the healthy vessel. Any other pretreatment procedures prior to balloon angioplasty were conducted at the investigator’s discretion. Study device sizing, introduction, positioning, and deployment were performed in compliance with the instructions for use. Postdilation was performed following stent deployment to confirm the vessel wall apposition of the device. Following the final implantation, a postprocedural angiogram to assess technical results was performed. Use of closure devices and postprocedural medication regimen was per investigator discretion, although dual antiplatelet therapy (DAPT) was recommended for a minimum of 6 months with the continuation of aspirin monotherapy through study follow-up. The follow-up regimen consisted of clinical evaluation at 30 days and clinical and imaging evaluation at 12, 24, and 36 months. Follow-up duplex ultrasound evaluation was performed by VASCORE (the Vascular Ultrasound Core Laboratory).

### End points and definitions

The primary effectiveness end point was PP at 12 months, and the primary safety end point was device- and procedure-related serious adverse events (SAEs) occurring within 30 days of the index procedure. A clinical events committee reviewed and adjudicated safety event seriousness and relationship to index procedure or device. Other end points assessed include acute procedural success (defined as successful delivery of the study device and complete coverage of the target lesion with no device- or procedure-related SAEs occurring prior to hospital discharge), primary-assisted and secondary patency, freedom from TLR (fTLR), freedom from amputation, improvement in the ankle-brachial index (ABI), change in Rutherford category, and assessment of stent fracture. Primary, primary-assisted, and secondary patency were assessed using standard definitions.^11^ TLR was defined as any repeat intervention in the study device, or within 5 mm proximally or distally to the device, to maintain, or restore patency. Tosaka class was defined as class I (focal ISR lesions ≤50 mm), class II (diffuse ISR lesions >50 mm), and class III (totally occluded ISR).^1^

### Data management and statistical analysis

Categorical data are presented as a count (percentage), and continuous data are presented as mean ± SD. Kaplan–Meier analysis was used to estimate patency rates and fTLR. SEs were <10% at all time points of end points. Any SEs >10% are indicated in the figures. A log-rank test was conducted to compare Kaplan–Meier curves among subgroups when analyzing patency and fTLR. In addition, a paired *t* test was used to compare ABI change from baseline. A *P* value of <.05 was considered statistically significant. Statistical analysis was performed using SAS version 9.4 (SAS Institute).

## Results

### Study population

**Figure 1:**
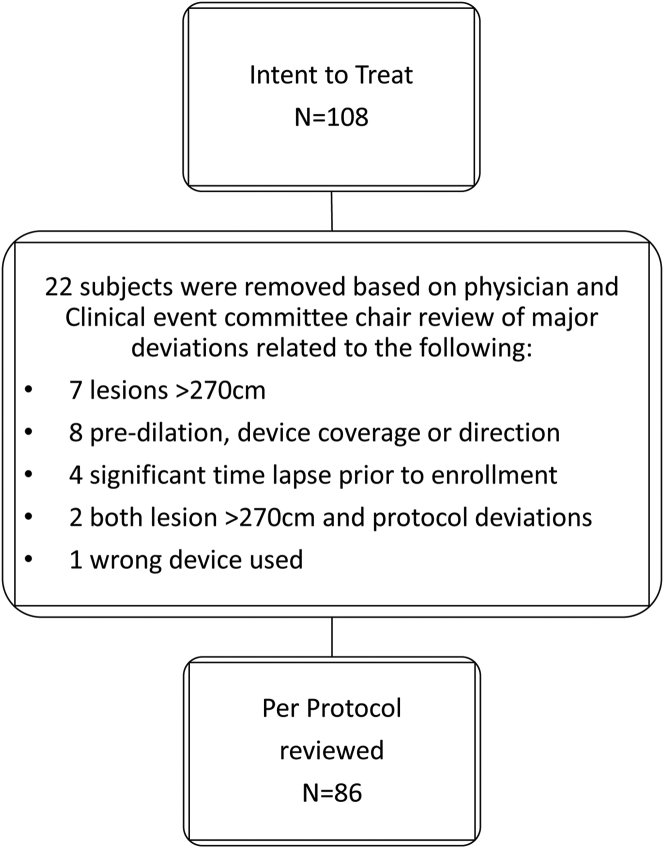
**Patient flowchart**.

The RELINE MAX study enrolled 108 patients from October 2015 to April 2018 in the United States and Europe. Twenty-two patients were removed from the intent-to-treat population because of procedural/treatment deviations and/or enrollment outside of eligibility criteria (Figure 1). Exclusions were reviewed and adjudicated by the global investigator and clinical events committee chairperson. Eighty-six patients were included for analysis (mean age, 70.9 ± 10.4 years), 77 (89.5%) presented with claudication (Rutherford categories 2-3), and 9 (10.5%) presented with chronic limb-threatening ischemia (Rutherford categories 4-5). Sex distribution was nearly equivalent, with 42 (48.8%) female patients. Core laboratory means lesion length was 12.4 ± 6.92 cm, and 25 patients (29.1%) presented with total occlusion. For the majority of patients, 66 (76.7%), it was the first reintervention to treat ISR. Baseline and clinical characteristics are presented in Table 2.

**Table 2:** Patient demographics and clinical characteristics.

Characteristic	N = 86
Male	44 (51.2)
Age, y	70.9 ± 10.4
Comorbidities	
Diabetes	46 (53.5)
Hypertension	73 (84.9)
Hyperlipidemia	68 (79.1)
Hypercholesterolemia	37 (43.0)
CAD	45 (52.3)
Smoking history	
Current	24 (27.9)
Former	44 (51.2)
Never	18 (20.9)
Previous interventions	
0	66 (76.7)
1	15 (17.4)
2	2 (2.3)
3	2 (2.3)
≥3	1 (1.2)
Previous minor amputation^a^	
No previous amputation	80 (93.0)
Minor amputation^a^	6 (7.0)
Rutherford category	
2	8 (9.3)
3	69 (80.2)
4	1 (1.2)
5	8 (9.3)
Resting ABI	0.68 ± 0.178
Resting TBI	0.425 ± 0.163
Lesion length, cm (N = 82)^b^^,^^c^	12.4 ± 6.9
<20 cm	69 (84.1)
≥20 cm	13 (15.9)
Percent stenosis, % (N = 61)^b^	69.2 ± 13.5
Total occlusion^b^	25 (29.1)
Moderate/severe calcification	28 (32.6)
Patent anterior tibial vessel	56 (65.1)
Patent posterior tibial vessel	53 (61.6)
Patent peroneal vessel	72 (83.7)

Values are n (%) or mean ± SD.

ABI, ankle-brachial index; CAD, coronary artery disease; TBI, toe-brachial index.

a Amputations include toe and transmetatarsal amputation.

b Core laboratory reporting.

c Core laboratory could not determine lesion length on 4 patients.

### Procedural characteristics

Core laboratory mean stented length was 19.5 ± 7.66 cm, and the majority (66.3%) of lesions were treated with one device (Table 3). Prior to the placement of the VIABAHN endoprosthesis, the following adjunctive procedures occurred: 21 laser atherectomy, 14 cutting/scoring balloons, 6 rotational atherectomy, 6 drug-coated balloon (DCB), 5 directional atherectomy, 2 PTA, and 1 thrombectomy and intravascular ultrasound. Acute procedural success was achieved in 85/86 (98.8%). One patient (1.2%) had procedure-related SAEs, including an arterial puncture site hematoma, transient hypotension, and moderate retroperitoneal bleeding. The postprocedural medication regimen was per the discretion of the treating physician; however, DAPT for a minimum of 6 months was recommended. During days 0 to 22, 92% were known to be on DAPT and 8% on a single antiplatelet or anticoagulant therapy.

**Table 3:** Treatment characteristics.

Characteristic	N = 86
Procedure time, min	66.0 ± 31.8
Femoral access	
Ipsilateral antegrade	21 (24.4)
Contralateral retrograde	65 (75.6)
Number of vessels patent prior to Procedure	
1 vessel	25 (29.1)
2 vessels	27 (31.4)
3 vessels	34 (39.5)
Predilatation successful	84 (97.7)
Number of devices implanted	
1	57 (66.3%)
2	27 (31.4%)
3	2 (2.3%)
Stented length, cm^a^	19.5 ± 7.66
Successful stent coverage	86 (100)
Postdilatation	84 (97.7)
Postprocedural residual stenosis ≤30%	84 (97.7)
Additional procedure required	11 (12.8)
Final tibial runoff	
0	0 (0.0)
1	20 (23.3)
2	37 (43.0)
3	29 (33.7)
Resting ABI at discharge	0.948 ± 0.129
Resting TBI at discharge	0.600 ± 0.329
Acute procedural success	
Yes	85 (98.8)
No	1 (1.2)
Index procedure-related SAEs	1 (100)

Values are n (%) or mean ± SD.

ABI, ankle-brachial index; SAEs, serious adverse events; TBI, toe-brachial index.

a Core laboratory reporting.

### Follow-up and safety outcomes

The number of patients completing the 30-day and 12-month visits was 82/86 (95.3%) and 66/78 (84.6%), respectively. Fifty (84.7%) of 59 and 47 (92.2%) of 51 patients returned for the 2- and 3-year follow-up visits, respectively. Of the 39 patients who withdrew from the study, 6 withdrew consent, 4 were withdrawn by the investigator, 10 died (none were device- or procedure-related), 9 required a surgical bypass of the study device (one subject had experienced acute limb ischemia [ALI]), 2 were lost to follow-up, and 8 withdrew for other reasons (4 were unavailable or unable to attend the 36-month follow-up, 2 moved out of state, and 2 did not return due to site closure).

Freedom from SAEs at 30 days was 83 (96.5%) of 86. No device-related SAEs were reported through 30 days. Nine procedure-related SAEs were reported in 3 patients through 30 days.

### Effectiveness outcomes at 12, 24, and 36 months

**Figure 2:**
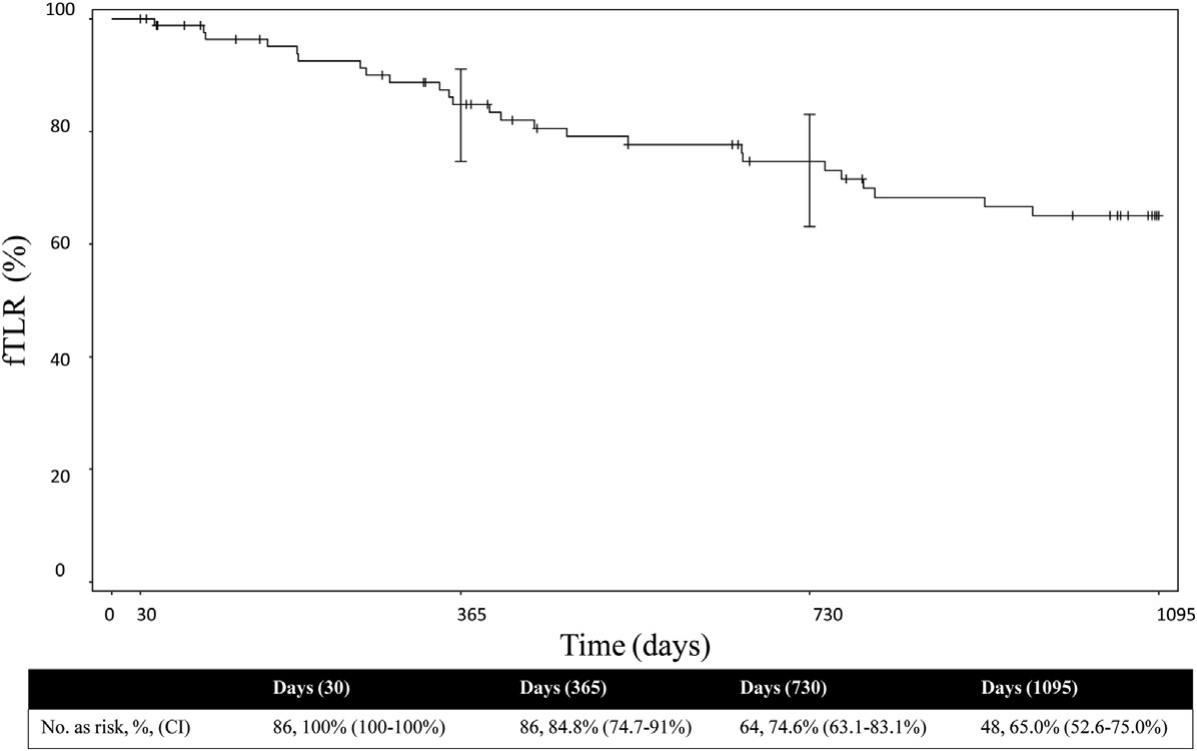
**Kaplan–Meier analysis for 36-month freedom from target lesion revascularization**.

At 12, 24, and 36 months, PP was 74.7%, 55.9%, and 44.7%, primary-assisted patency was 80.4%, 64.6%, and 56.4%, and secondary patency was 98.4%, 89.4%, and 82.3%, respectively (Central Illustration). Freedom from TLR at 12, 24, and 36 months was 84.8%, 74.6%, and 65.0%, respectively (Figure 2). Reason for reinterventions included 82% because of occlusion, 14% had peak systolic velocity ratio >2.5, and 4% were because of claudication. A total of 97% of subjects were free from ALI over 3 years. Freedom from major amputation was 100% through 36 months. Per core laboratory assessment, no stent fractures were observed through 36 months.

**Central Illustration:**
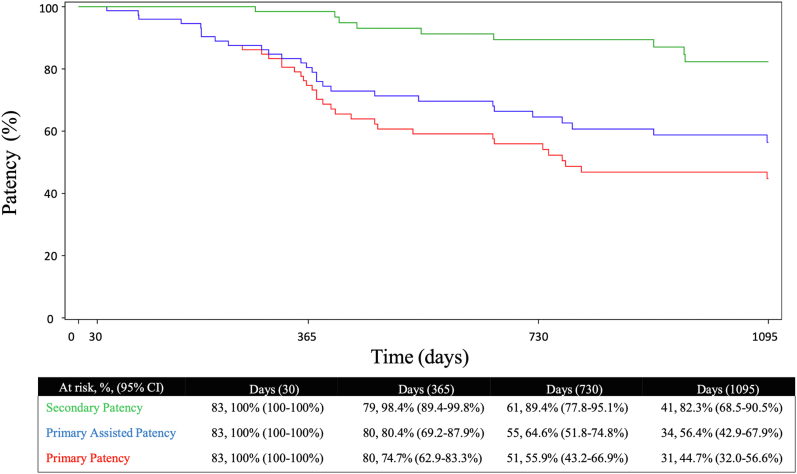
**Kaplan–Meier analysis of primary, primary-assisted, and secondary patency at 12, 24 and 36 months**.

**Figure 3:**
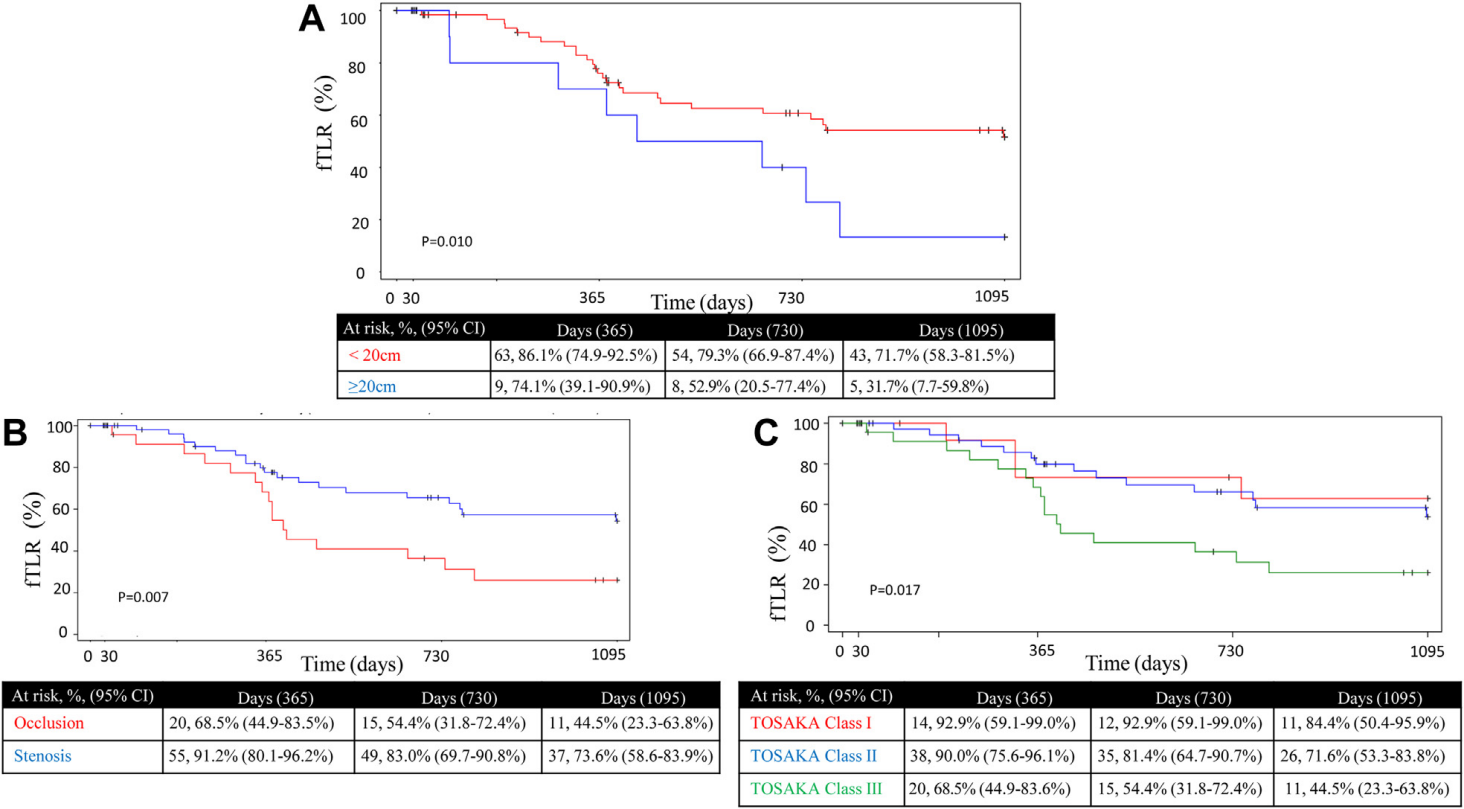
**Kaplan–Meier analysis freedom from target lesion revascularization stratified by anatomic characteristics**: (**A**) lesion length; (**B**) disease severity; (**C**) Tosaka classification. fTLR, freedom from target lesion revascularization.

Stratified analysis of PP through 36 months found significant differences associated with lesions ≥20 cm (*P* = .041), occlusive vs stenotic lesions (*P* = .016), and Tosaka I vs II vs III (*P* = .031). For fTLR, the same characteristics were found to be significant: lesion length ≥20 cm (*P* = .010), occlusive vs stenotic lesions (*P* = .007), and Tosaka I vs II vs III (*P* = .017) (Figure 3). There was no statistically significant association between PP and fTLR, lesion calcification, diabetes mellitus, Rutherford category, sex, number of devices, smoking status, or runoff vessels. Lesion length was the only analyzed characteristic determined to be associated with a statistically significant difference in secondary patency rates (*P* < .001). Patients with lesions <20 cm had a 3-year secondary patency of 87.6%, whereas those with ≥20 cm had a 3-year secondary patency of 58.3%.

### Change in ABI and Rutherford categories

**Figure 4:**
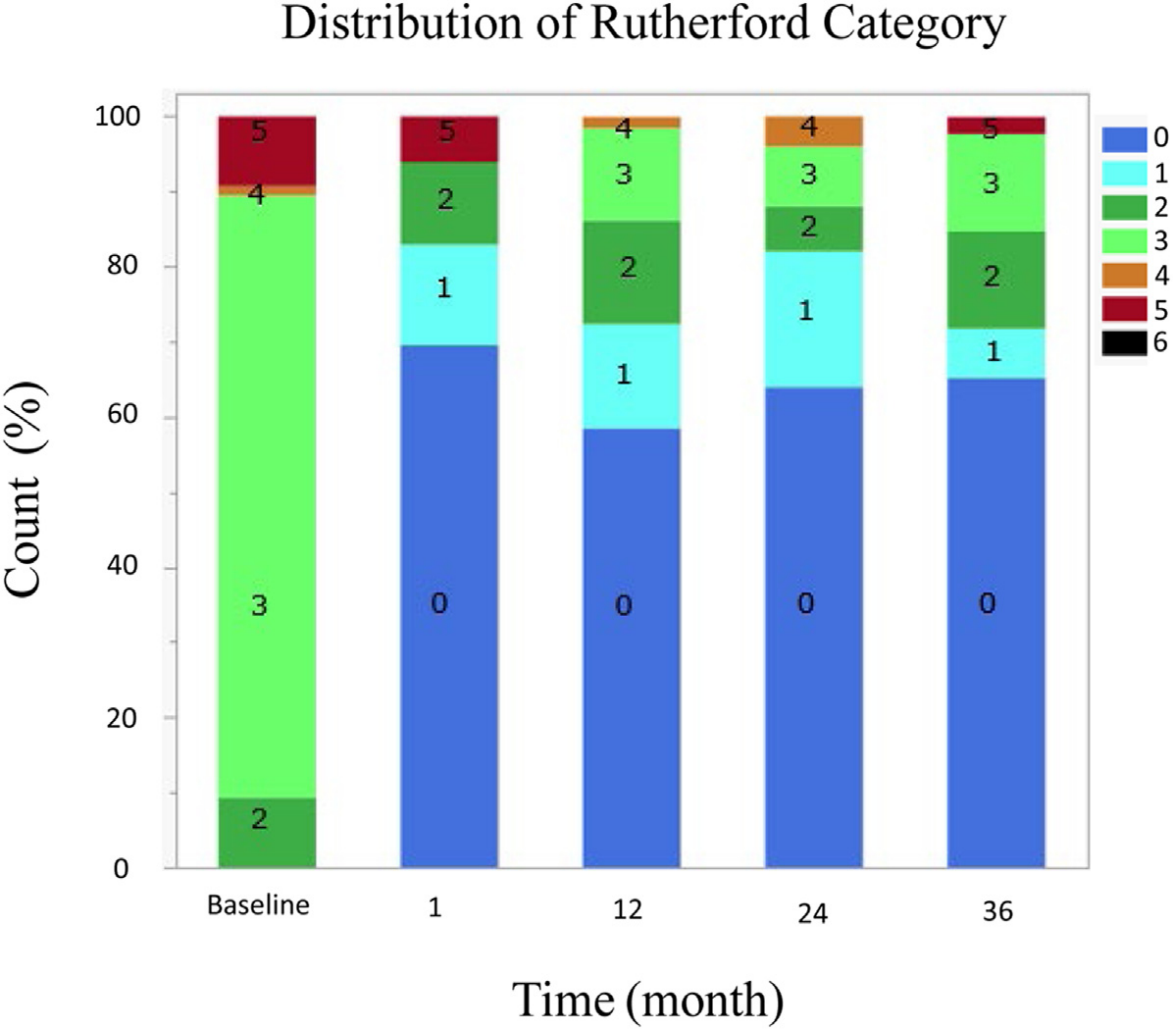
**Distribution of Rutherford categories**.

At 30 days, 12, 24, and 36 months follow-up, the mean change improvement in ABI from baseline (0.68 ±.178) assessment was .329, .238, .212, and .243, respectively (*P* < .001). Improvement in the Rutherford category by ≥1 category was observed in 92.7% of patients at 30 days, 87.7% at 12 months, 86.0% at 24 months, and 80.4% at 36 months. The distribution of the Rutherford category assessment is displayed in Figure 4.

## Discussion

This study demonstrated the safety and effectiveness of the VIABAHN endoprosthesis for treating bare metal ISR. At the 1-, 2-, and 3-year time points, PP was 74.7%, 55.9%, and 44.7%, primary-assisted patency was 80.4%, 64.6%, and 56.4%, and secondary patency was 98.4%, 89.4%, and 82.3%, respectively. Freedom from TLR was 84.8%, 74.6%, and 65.0% at 1, 2, and 3 years, respectively, with 9 patients converting to bypass through 36 months, which were included in the patency calculations. No major amputations or device fractures occurred.

With limited quality data associated with the complexity of the vessel bed and lesion formation, the treatment of SFA-ISR is often challenging; durable treatment options are required to support this patient population and to reduce further interventions. Robust evidence supports that PTA is not an effective treatment in these lesions and, thus, should no longer be ethically selected as a comparator in clinical trials.^12^

Several SFA-ISR treatment modalities (ie, laser photoablation, cryoplasty, directional atherectomy, etc.) have been investigated, but most are not comparable because of short follow-up times. In this study, PP at 1, 2, and 3 years was 74.7%, 55.9%, and 44.7%, respectively, whereas other studies using alternative treatments had lower 1-year PP rates ranging from 25% to 37.8%,^13^^,^^14^ and comparable 2-year rate of 53.8%.^15^ In this study, we demonstrate fTLR results of 84.8%, 74.6%, and 65.0% at 1, 2, and 3 years, respectively, whereas studies on alternate therapies showed lower fTLR at 1 year of 66.8%^16^ and at 2 years of 66%.^17^ The safety and efficacy of directional atherectomy with the SilverHawk device were evaluated in FP-ISR with mean lesions of 10.8 cm and a reported 1-year PP rate of 25%.^14^ The EXCITE ISR trial data suggest that while safe and effective, laser atherectomy for FP-ISR showed poor results, including a 6-month fTLR of 73.5% and a low PP rate of approximately 35% at 1 year.^9^

Paclitaxel has shown contradictory outcomes. The ISR subset analysis of in the IN.PACT global registry study had a mean lesion length of 17 cm at 12 months PP was 88.7%, with a drop to 80.7% observed after 13 months.^18^ The Schmidt et al^19^ 2016 IN.PACT DCB study had 1- and 2-year PP rates of 76.6% and 48.6%, a 2-year fTLR of 58.7%, and a 2.1% rate of amputations, respectively. In other IN.PACT studies, fTLR was reported at 86% at 1 year and 60% at 3 years, and a 1-year SAEs rate of 15.9% and 2% rate of amputation.^7^^,^^20^ Also, decreased PP was reported in the randomized Paclitaxel Balloon Versus Standard Balloon in In-stent Restenoses of the Superficial Femoral Artery (PACUBA) trial when patients were treated with DCB (40.7%) vs conventional PTA (13.4%) at 12 months.^21^ The long-term negative outcome is observed in the DEBATE-ISR study, a study assessing DCB vs PTA in patients with diabetes, which found that TLR was significantly lower in DEBs^22^ at 1 year, but rates were similar at 3 years.^7^ Delayed restenosis after DCB could imply an initial inhibition of neointimal hyperplasia formation followed by disease progression. The PP of the Zilver PTX at 1 year was 78.8%.^8^ Sugimoto et al^23^ reported 1, 2, and 3-year fTLR rates of 85.8%, 79.5%, and 78.1%, respectively, which are similar to our data. In comparison with Sugimoto et al,^23^ Rutherford category changes were comparable at 1 and 3 years and better at the 2-year time point. Through 3 years, no fractures were observed compared with a 1.2% Zilver PTX fracture rates through 1 year and 0.8% and 2.5% through 1 and 5 years, respectively.^8^^,^^23^

In the first publication, which studied the VIABAHN endoprosthesis exclusively in SFA-ISR lesions, an excellent PP was reported at 1 and 3 years of 85.1% and 81.4%, respectively.^24^ With 37% of the population presenting with chronic limb-threatening ischemia, a mean lesion length of 24.5 cm, and 52% chronic total occlusions, the authors point to rigorous adherence to operative best practices and administration of long-term dual DAPT as key factors responsible for the favorable outcomes observed; laser atherectomy was also used in a third of patients (typically those presenting with chronic total occlusions and long lesions). See Table 4 to view comparison data for fTLR.^25^

**Table 4:** Comparison of in-stent restenosis studies.

Reference, y	ISR subjects	Treatment	Lesion length (cm)	Occlusions (%)	CLTI (%)	Tosaka II/III (%)	fTLR – 1 y (%)	fTLR – 2 y (%)	fTLR – 3 y (%)
RELINE MAX per protocol	86	GORE VIABAHN	12.4	29.1	10.5	81.9	84.8	74.6	65.0
RELINE MAX ITT	108	GORE VIABAHN	13.9	33.3	13.0	81.9	82.8	74.6	66.8
Bosiers et al,^25^ 2020	39	GORE VIABAHN	17.3	23.1	12.8	-	80.2	66.3	-
Schmidt et al,^13^ 2014	90	Turbo Elite Laser atherectomy	12.3	30 (34.1)	3.8	73.3	64.4	-	-
Trentmann et al,^14^ 2010	33	Silver HawkTM direct atherectomy	10.8	7 (20)	26	-	-	-	-
Böhme et al,^15^ 2021	31	DCB	15.9	8 (25.8)	12.9	-	87.1^c^	72.0	-
Shammas et al,^16^ 2013	60	JetStream XC atherectomy	19.9	33 (55)	12	74	66.8	-	-
Kokkinidis et al,^17^ 2020	66	Turbo Elite Laser atherectomy + DCB	26.3	49 (74)	27	100	-	66	45
Dippel et al,^9^ 2015	169	EXCImer Laser + PTA	19.6	30.5	16	-	-	-	-
Brodmann et al,^18^ 2017	131	IN.PACT Admiral DCB	17.2	34.0	9.2	-	92.7^c^	-	-
Schmidt et al,^19^ 2016	107	DCB	24.0^a^	-	-	-	83	58.7	-
Virga et al,^20^ 2014	39	IN.PACT Admiral DCB	-	-	-	79.5	∼92^b^	78.4	-
Grotti et al,^7^ 2016 Liistro et al,^22^ 2014	44	IN.PACT Admiral DCB	13.2	-	75	85	∼86^b^	∼68^b^	60
Kinstner et al,^21^ 2016	35	FREEWAY DCB	17.3	11 (31)	0	77	49.0^c^	-	-
Zeller et al,^8^ 2013	119	Zilver PTX	13.3	31.1	-	-	81.0	60.8	
Sugimoto et al,^23^ 2021	177	Zilver PTX	17.8	35.3	22.3	-	85.8	79.5	78.1

CLTI, chronic limb-threatening ischemia; DCB, drug-coated balloon; fTLR, freedom from target lesion revascularization; ISR, in-stent restenosis.

a Includes 181 non-ISR subjects.

b Estimate based on figures.

c Clinically driven fTLR.

The multicenter European RELINE study was the first randomized trial assessing the VIABAHN endoprosthesis as a treatment for SFA-ISR.^10^ At 12 months, PP was 74.8% for the VIABAHN endoprosthesis compared with 28.0% with PTA, and only 1 patient (2.5%) experienced a device-related adverse event in the VIABAHN endoprosthesis group through 30 days. The postmarket RELINE MAX study was designed to assess whether these outcomes would be duplicated in the US patient population and follow-up through 36 months. Our outcomes have been remarkably consistent with those observed in the RELINE trial. We report freedom from device- or procedure-related adverse events of 96.5% at 30 days and PP of 74.7% at 12 months.

Post hoc subset analyses were performed for factors believed to impact patency outcomes, such as lesion length, sex, disease severity, vessel runoff, diabetes, and degree of calcification. Our analysis found that long lesions ≥20 cm, the presence of occlusive disease, and higher Tosaka class negatively impacted PP and fTLR. These findings highlight the technical difficulty of treating complex ISR disease. Other factors, such as calcification level, diabetes mellitus, Rutherford category, sex, number of devices, smoking status, and runoff vessels, did not impact patency or fTLR outcomes. In this study, patients with moderate and severe calcification had better outcomes (although not significant) than those with mild calcification, in contrast to Schmidt et al,^19^ which found that severe calcification and diabetes were negative predictors, and the study showed PP after DCB treatment for ISR treatment of 48.6%, freedom from TLR of 58.7% at 2 years, and major amputation rate of 2.1% at 2 years. Our results further demonstrate that these risk factors, often associated with complex patient populations, were not associated with fTLR through 3 years as compared with DCB, where diabetes, calcification, and female gender increase the risk of restenosis.^19^

In our experience, a good clinical outcome with the VIABAHN endoprosthesis is largely attributable to adequate vessel preparation and procedural execution. Stent-graft coverage should extend into a healthy vessel, and as multiple studies have demonstrated, aggressive oversizing (>20%) should be avoided.^26^, ^27^, ^28^ Although not assessed in this study, it is the opinion of the authors that the use of intravascular ultrasound and quantitative angiography are instrumental in achieving meticulous vessel measurement and device sizing and should serve as a focal point for future studies in this area. Ensuring good vessel inflow and outflow through the stent graft at the time of procedure is also of the utmost clinical significance; good flow dynamics attained in at least 1 runoff vessel should be sufficient to achieve a favorable outcome. Postprocedure, patients should be on DAPT for a minimum of a year; if possible, lifelong DAPT is preferable. In our experience, if endoprosthesis occlusion does occur, it has largely not been associated with ALI. Twenty-eight patients needed reintervention, and 97% of patients were free of ALI through 36 months in this challenging SFA-ISR population. These data are comparable with SFA VIABAHN endoprosthesis treatment in de novo SFA disease with 1 ALI and no ALI reported through 2 and 5 years.^26^, ^27^, ^28^ The findings demonstrate that there is no significantly increased risk for ALI, a long-standing point of debate with covered stent grafts. We recommend vigilant duplex ultrasound surveillance after stent-graft implantation, with aggressive reintervention for any observed restenosis (peak systolic velocity ratio >2.5), even if asymptomatic.

### Study limitations

Almost all subset analyses of PP involved comparison in which 1 subgroup was substantially smaller, limiting the robustness of the data. This study was not randomized to a comparator group, and the medication regimen was left to the physician’s discretion, although all patients were prescribed DAPT for at least 1 year. The patient sample size was relatively small, with only 69% of patients who returned for x-ray evaluation.

### Conclusion

The VIABAHN endoprosthesis is safe and effective in treating long and complex ISR lesions. PP outcomes up to 36 months were similar regardless of the degree of calcification, number of runoff vessels, or sex. No stent-graft fractures and no major amputations occurred through 36 months.
